# Environment And Genetics in Lung cancer Etiology (EAGLE) study: An integrative population-based case-control study of lung cancer

**DOI:** 10.1186/1471-2458-8-203

**Published:** 2008-06-06

**Authors:** Maria Teresa Landi, Dario Consonni, Melissa Rotunno, Andrew W Bergen, Alisa M Goldstein, Jay H Lubin, Lynn Goldin, Michael Alavanja, Glen Morgan, Amy F Subar, Ilona Linnoila, Fabrizio Previdi, Massimo Corno, Maurizia Rubagotti, Barbara Marinelli, Benedetta Albetti, Antonio Colombi, Margaret Tucker, Sholom Wacholder, Angela C Pesatori, Neil E Caporaso, Pier Alberto Bertazzi

**Affiliations:** 1 Division of Cancer Epidemiology and Genetics, National Cancer Institute, NIH, Bethesda, MD, USA; 2 EPOCA, Epidemiology Research Center, University of Milan, and Fondazione IRCCS Ospedale Maggiore Policlinico, Mangiagalli e Regina Elena, Italy; 3 Division of Cancer Control and Population Sciences, National Cancer Institute, NIH, Bethesda, MD, USA; 4 Center for Cancer Research, National Cancer Institute, NIH, Bethesda, MD, USA

## Abstract

**Background:**

Lung cancer is the leading cause of cancer mortality worldwide. Tobacco smoking is its primary cause, and yet the precise molecular alterations induced by smoking in lung tissue that lead to lung cancer and impact survival have remained obscure. A new framework of research is needed to address the challenges offered by this complex disease.

**Methods/Design:**

We designed a large population-based case-control study that combines a traditional molecular epidemiology design with a more integrative approach to investigate the dynamic process that begins with smoking initiation, proceeds through dependency/smoking persistence, continues with lung cancer development and ends with progression to disseminated disease or response to therapy and survival. The study allows the integration of data from multiple sources in the same subjects (risk factors, germline variation, genomic alterations in tumors, and clinical endpoints) to tackle the disease etiology from different angles. Before beginning the study, we conducted a phone survey and pilot investigations to identify the best approach to ensure an acceptable participation in the study from cases and controls. Between 2002 and 2005, we enrolled 2101 incident primary lung cancer cases and 2120 population controls, with 86.6% and 72.4% participation rate, respectively, from a catchment area including 216 municipalities in the Lombardy region of Italy. Lung cancer cases were enrolled in 13 hospitals and population controls were randomly sampled from the area to match the cases by age, gender and residence. Detailed epidemiological information and biospecimens were collected from each participant, and clinical data and tissue specimens from the cases. Collection of follow-up data on treatment and survival is ongoing.

**Discussion:**

EAGLE is a new population-based case-control study that explores the full spectrum of lung cancer etiology, from smoking addiction to lung cancer outcome, through examination of epidemiological, molecular, and clinical data. We have provided a detailed description of the study design, field activities, management, and opportunities for research following this integrative approach, which allows a sharper and more comprehensive vision of the complex nature of this disease. The study is poised to accelerate the emergence of new preventive and therapeutic strategies with potentially enormous impact on public health.

## Background

Lung cancer, the largest single cause of cancer mortality in the U.S. and worldwide, kills more people every year than cancers of the breast, prostate and colon combined. The SEER registry estimated that 213,380 new cases and 160,390 deaths would occur in the US in 2007 [1]; age-standardized incidence rates across European countries in 2005 [2] varied between 21 and 77 per 100,000 in men and 3 and 35 in women, with age-standardized mortality rates ranging between 32 and 52 per 100,000 in men and between 8 and 19 per 100,000 in women. Traditional approaches to treatment, screening and prevention of lung cancer are inadequate, and there is currently no effective chemoprevention or proven effective screening for this disease. Newer molecular targeted chemotherapeutic agents result in short-term improvements in survival in responsive subsets, but have had marginal impact on overall mortality.

Epidemiological and other scientific investigations have clearly implicated tobacco smoking as the primary cause of lung cancer, and yet the precise molecular alterations induced by smoking in lung tissue that lead to lung cancer and impact survival have remained obscure. Many lines of evidence consistently support a hereditary influence on lung cancer risk, with polygenic mechanisms and complex interactions, including epistatic relationships [3]. Over 100 studies have examined individual candidate genes in relation to lung cancer during the last decade. Despite this body of literature, no clear consensus exists on the role these factors play in influencing lung cancer susceptibility. The vast majority of published studies in this field have been underpowered in relation to the realistic main and interactive effects suggested by recent metanalyses [4-6]. In addition, they have been focused only on a few genes or few risk factors, whereas many genes and interactions between genes and other risk factors are likely important for lung carcinogenesis.

We designed a large population-based case-control study precisely to combine a traditional molecular epidemiology design with a more *integrative *approach to investigate the effects of a dynamic process that begins with smoking initiation, proceeds through dependency and smoking persistence, continues with lung cancer development and ends with progression to disseminated disease or response to therapy and survival.

This approach maximizes the use of epidemiological, clinical, behavioral, and molecular data from the same subjects to answer multiple questions at the same time. For example, we supplemented the detailed information on smoking and family history of lung cancer needed for the etiologic study with additional information on behavioral factors and family history of smoking from the first degree relatives of cases and controls. This allows study of genetic determinants of persistence or cessation of smoking in controls and enhances our ability to assess the effect of shared familial tobacco use on the putative genetic effect of family history of lung cancer on lung cancer risk. We supplemented collection of blood specimens needed to study genetic variation on susceptibility to lung cancer with fresh frozen lung tissue samples to study whether these germ-line variations (with or without smoking exposure) are associated with altered expression of these same genes and related pathways in the target organ. We supplemented detailed pathology information from each lung cancer case needed to assess etiologic heterogeneity with collection of treatment and outcome data to learn about disease progression and efficacy of treatment.

The Environment And Genetics in Lung cancer Etiology (EAGLE) study is an integrative population-based study of lung cancer. We present here the study design, field activities, management organization, strategies we used to increase the participation rate in population controls, and characteristics of the enrolled subjects. We also provide our perspectives on how the integration of complex epidemiological, clinical and genomic data and participation of multiple investigators can shed light on the etiology, prevention and treatment of this deadly disease.

## Methods

### Study Design

The study was conducted in the Lombardy region of Italy, which includes approximately 9,600,000 people and is served by a network of modern hospitals, medical schools, and a regional health service. The catchment area includes 5 cities (Milan, Monza, Brescia, Pavia, and Varese) and surrounding towns and villages, for a total of 216 municipalities, encompassing over 3,000,000 people. The theoretical ideal is random collection of cases from the population, e.g., through a cancer registry. However, random selection from hundreds of large and small hospitals would have made collection of biospecimens (particularly fresh frozen tissue samples) and detailed epidemiological and clinical data unfeasible. Thus, we designed EAGLE to be as close as possible to a really comprehensive population-based study through enrollment of cases in a defined set of hospitals, which examine approximately 80% of all lung cancer cases from the catchment area. These hospitals were selected based on a review of the hospital admission/discharge records from the years 1997–2000.

EAGLE includes 2101 verified, incident, primary lung cancer cases of any histologic type, with the exception of carcinoids, and 2120 healthy population-based controls. Participants are both male and female, born in Italy, of Italian nationality, and with official residence in the 216 selected municipalities, at ages between 35 and 79 years old at diagnosis (cases) or enrollment for interview (controls) that signed an informed consent form to participate in the study. The study was approved by the Institutional Review Board (IRB) of each participating hospital and university in Italy and by the National Cancer Institute, Bethesda, MD.

EAGLE's study size is powered to detect small increases in risk for factors with moderate frequency; for example the power is at least 80% to detect an association between a given genotype and lung cancer risk with an OR of 1.4 for at-risk genotype frequency between 10% and 90%. Under a multiplicative gene-environment interaction model, the study is large enough to reject at a 0.05-level with 80 percent power an interaction of 0.5 between the highest smoking category relative to the non-smoking category when the at-risk genotype frequency is > 13%, and of 0.2, if the at-risk genotype frequency is 5% or higher and the distributions of smoking and the gene are independent [7].

### Lung cancer cases

We recruited cases from thirteen hospitals. A detailed description and link to the respective hospitals is available on the EAGLE website [8]. The first diagnosis of all lung cancer cases occurred in the period between April 22^nd^, 2002 and February 28^th^, 2005, and enrollment continued until June 30^th^, 2005. Two research physicians per hospital reviewed daily hospital admission logs in different departments, identified cases of suspected lung cancer in the specified age range and from the catchment area, and arranged for collection of blood specimens and for subject interview. Rapid communication with the central institute from each hospital was performed using a web-based case-registry connected through dedicated Integrated Services Digital Network (ISDN) lines. Reasons for non-eligibility were recorded for all subjects. Subjects who declined to participate were asked to answer a few questions on smoking and demographic characteristics that allowed us to obtain a more comprehensive picture of the lung cancer cases of the area within the study period.

The diagnosis of lung cancer was established based on clinical criteria and confirmed by pathology reports from surgery, biopsy or cytology samples in approximately 95% of cases, and on clinical history and imaging for the remaining 5%. The date of diagnosis was defined as the date of the first clinical study to report a suspicious lesion (for example, chest X-ray or CT-scan) that led directly to diagnosis. To verify the diagnosis, we examined the clinical history, bronchoscopy and biopsy results, and x-ray and thorax CT scans (and MRI or PET scans when available) and hospital discharge letter for each case. In addition, we reviewed surgery descriptions and pathology reports for the surgical cases, and biopsies and/or cytology reports from brushing, broncho-alveolar lavage, sputum, bronchoaspirate, or pleural or pericardial effusion for the non-surgical cases. All available imaging documenting lymph node and/or distant metastases or other functional/clinical conditions that excluded surgery were also assessed. Tumor histology was coded according to the WHO Histological Typing of Lung and Pleural Tumors (1999); clinical and/or post-surgical staging was performed according to the International System for Staging Lung Cancer adopted by the American Joint Committee on Cancer and the Union Internationale Contre le Cancer [9]. To verify extra-thorax metastases, we reviewed abdominal CT scans and ultrasounds, brain CT scans or MRI, and bone scintigraphy scans. To standardize diagnostic criteria across hospitals we reviewed clinical documentation and when necessary made changes to the original diagnosis/staging; in these instances the reason and the specific changes made were documented in a decision log. Diagnoses from approximately 10% of cases were reviewed and confirmed by an experienced independent pulmonary pathologist from the National Cancer Institute, NIH (Dr. Ilona Linnoila).

At study completion, we had screened 4630 subjects of whom over 2706 were eligible based on the inclusion criteria. We enrolled 2343 cases (86.6% of the eligible cases), of whom 179 (7.6%) were determined not to have lung cancer after diagnosis review; an additional 63 subjects had an uncertain diagnosis or were determined not to fit the inclusion criteria. Thus, subjects with confirmed diagnosis and characteristics fitting the inclusion and exclusion criteria were 2101. Epidemiological data and DNA specimens were collected from 98.4% and 97.3% of cases, respectively (Table 1).

**Table 1:** Distribution of questionnaire data and biological samples in cases and controls from EAGLE

	**Cases No. 2101**		**Controls No. 2120**	
		
	No.	%	No.	%
With data on major risk factors	2067	98.4	2116	99.8
With data on diet and behavioural factors	1903	90.6	2073	97.8
With at least one DNA sample*	*2045*	*97.3*	*2117*	*99.9*
With blood sample	1891	90.0	1841	86.8
With buccal rinse sample	154	7.3	282	13.3
*With at least one pathology sample*	*1212*	*57.7*		
With tissue slides	1192	56.7		
With paraffin-embedded tissue blocks	656	31.2		
With fresh tissue samples	436	20.8		

*Two cases and six controls donated both blood and buccal samples

The distribution of EAGLE lung cancer cases by area of residence, gender and age (matching variables) is reported in Table 2; the distribution by cigarette smoking, histology and stage is reported in Tables 3A and 3B for females and males, respectively.

**Table 2:** Population in the catchment area* and distribution of EAGLE subjects by gender, age, and residence (matching variables)

	**Population**		**Cases**		**Controls**	
			
	**No.**	**%**	**No.**	**%**	**No.**	**%**
**Area**						
Brescia	272,786	16.6	275	13.1	248	11.7
Milan	963,341	58.7	1369	65.2	1440	67.9
Monza	155,491	9.5	144	6.8	117	5.5
Pavia	122,036	7.4	137	6.5	130	6.1
Varese	128,420	7.8	176	8.4	185	8.7
**Gender**						
Females	867,310	52.8	449	21.4	500	23.6
Males	774,764	47.2	1652	78.6	1620	76.4
**Age**						
35–39	242,876	14.8	12	0.6	17	0.8
40–44	203,923	12.4	19	0.9	28	1.3
45–49	185,568	11.3	52	2.5	71	3.3
50–54	206,127	12.6	133	6.3	127	6.0
55–59	183,214	11.2	240	11.4	300	14.1
60–64	196,734	12.0	359	17.1	371	17.5
65–69	167,764	10.2	474	22.6	489	23.1
70–74	143,191	8.7	471	22.4	424	20.0
75–79	112,677	6.9	341	16.2	293	13.8
**Total**	1,642,074	100.0	2101	100.0	2120	100.0

*N = 216 municipalities, 2001 census; age 35–79 years.

**Table 3:** Distribution of EAGLE lung cancer cases by cigarette smoking*, histology and stage in females and males.

**A – Females**										
	**Never**		**Former**		**Current**		**Missing**		**Total**	
					
	**No.**	**%**	**No.**	**%**	**No.**	**%**	**No.**	**%**	**No.**	**%**

**Histology**										
Non small cell lung carcinoma (NSCLC)	101	87.8	96	78.0	146	73.0	10	90.9	353	78.6
Adenocarcinoma	85	73.9	64	52.0	90	45.0	5	45.4	244	54.3
Squamous cell	7	6.1	19	15.4	22	11.0	1	9.1	49	10.9
Large cell	2	1.7	6	4.9	12	6.0	0	-	20	4.5
NSCLC, not defined	7	6.1	7	5.7	22	11.0	4	36.4	40	8.9
Small cell carcinoma	3	2.6	8	6.5	29	14.5	0	0.0	40	8.9
Others (mixed types)	5	4.4	13	10.6	18	9.0	1	9.1	37	8.2
Missing	6	5.2	6	4.9	7	3.5	0	0.0	19	4.2
**Stage**										
0	0	0.0	0	0.0	0	0.0	0	0.0	0	0.0
IA	11	9.6	13	10.6	20	10.0	2	18.2	46	10.2
IB	14	12.2	13	10.6	23	11.5	0	0.0	50	11.1
IIA	0	0.0	3	2.4	3	1.5	1	9.1	7	1.6
IIB	10	8.7	10	8.1	14	7.0	0	0.0	34	7.6
IIIA	13	11.3	18	14.6	26	13.0	0	0.0	57	12.7
IIIB	18	15.6	15	12.2	28	14.0	3	27.3	64	14.3
IV	48	41.7	50	40.7	81	40.5	5	45.4	184	41.0
X	1	0.9	1	0.8	5	2.5	0	0.0	7	1.6

**Total**	115	100.0	123	100.0	200	100.0	11	100.0	449	100.0


**B – Males**										

	**Never**		**Former**		**Current**		**Missing**		**Total**	
					
	**No.**	**%**	**No.**	**%**	**No.**	**%**	**No.**	**%**	**No.**	**%**

**Histology**										
Non small cell lung carcinoma (NSCLC)	28	84.9	607	80.0	662	79.0	14	60.9	1311	79.4
Adenocarcinoma	20	60.6	283	37.3	305	36.4	6	26.1	614	37.2
Squamous cell	5	15.2	233	30.7	241	28.8	5	21.7	484	29.3
Large cell	0	0.0	23	3.0	33	3.9	1	4.4	57	3.5
NSCLC, not defined	3	9.1	68	9.0	83	9.9	2	8.7	156	9.4
Small cell carcinoma	1	3.0	73	9.6	100	11.9	2	8.7	176	10.7
Others (mixed types)	1	3.0	39	5.2	38	4.5	2	8.7	80	4.8
Missing	3	9.1	39	5.2	38	4.5	5	21.7	85	5.1
**Stage**										
0	0	0.0	1	0.1	0	0.0	0	0.0	1	0.1
IA	3	9.1	78	10.3	59	7.0	1	4.4	141	8.5
IB	1	3.0	109	14.4	93	11.1	2	8.7	205	12.4
IIA	0	0.0	11	1.4	11	1.3	0	0.0	22	1.3
IIB	4	12.1	65	8.6	76	9.1	1	4.4	146	8.8
IIIA	1	3.0	78	10.3	97	11.6	1	4.4	177	10.7
IIIB	4	12.1	102	13.5	150	17.9	3	13.0	259	15.7
IV	20	60.6	295	38.9	331	39.5	14	60.9	660	40.0
X	0	0.0	19	2.5	21	2.5	1	4.4	41	2.5

**Total**	33	100.0	758	100.0	838	100.0	23	100.0	1652	100.0

*Never smoking, Former smoking and Current smoking

### Population controls

The population with official residence in the catchment area represented the study pool from which controls were sampled. The Regional Health Service (RHS) database contains information on subjects' demographics and on the family physician for virtually all Italians. We sampled population controls from updated population databases obtained periodically (twice a year) from the Lombardy Region; the age of controls was calculated as of pre-specified dates, i.e., July 22^nd ^and January 22^nd ^of each year. Controls were selected randomly within 90 cells (see below) to yield a set of controls with a distribution that initially approximated the case distribution based on year 2000 lung cancer admissions, and subsequently was based on enrolled EAGLE lung cancer cases, for 3 key variables: residence (5 areas: Brescia, Milan, Monza, Pavia, Varese), gender, and five-year age classes in the range 35–79 years (5 × 2 × 9 combinations of residence-gender-age categories). To select controls, a random number was assigned to each subject using statistical software, and the records were sorted based on this random number. Each subject with a number below the target enrollment number for the individual's cell was selected to be invited into the study. The family physicians for the potential study subjects were identified. The selected physicians were then asked to provide information about eligibility of the potential study subjects and, if eligible, to contact the selected controls to inform them about the study. Eligible controls were contacted by letter from the study personnel, followed by a phone call. When the phone number for the selected individuals could not be found, we searched for the phone numbers of other members of the family identified through contact with the corresponding municipalities, or sent pre-stamped return-cards requesting contact information.

At study completion we had sent invitation letters followed by phone call to 3314 potential controls. Traced eligible subjects were 2774, of whom 2012 accepted to participate. Completion rate was 60.7% (subjects who accepted to participate/contacted subjects) and participation rate was 72.5% (subjects who accepted to participate/eligible subjects). Moreover, we sent pre-stamped return-cards to 393 subjects whom we were unable to trace through the phone. Of these, 155 were eligible, and 108 accepted to participate (completion rate = 27.5%, participation rate = 69.7%). Overall, we enrolled 2120 controls, with an overall participation rate of 72.4%. Epidemiological data and DNA samples were collected from 99.8% and 99.9% of controls, respectively (Table 1).

The distribution of EAGLE controls by area of residence, gender and age (matching variables) is reported in Table 2.

### Strategies to improve subjects' participation rate

The strategies we followed for subjects' enrollment described above were derived from a series of pilot studies we conducted before officially beginning the EAGLE field activities. Because determinants of participation in cases (medical condition, performance status) are different from the determinants in controls (altruism, time), we were concerned that lack of participation might affect estimates of genetic or environmental main effects and interactions on lung cancer risk [10-12]. We made concerted efforts to maximize participation of both cases and controls, and used several strategies and incentives to increase the participation rate in controls.

First, to verify the feasibility of a population-based study in the Lombardy region of Italy and study the characteristics related to potential subjects' participation, we conducted a phone survey of 1053 healthy subjects from the catchment area, selected from the rosters of the Regional Health Service, to have the age, gender and geographic distribution expected in the lung cancer cases. We asked the contacted subjects whether they would agree to participate in a study of lung cancer that would require an interview and donation of a blood sample. Only 320 (30%) of the subjects responded "Yes". There was only modest variation in response by municipality, age, gender, tobacco use, and educational level. From this effort, we obtained valuable information about preferred location for blood drawing, days of the week, and times most convenient for participation. This information was used in a series of pilot studies in which we first contacted the selected individuals by mail with follow-up by telephone, offering participation in the study at the closest hospitals of the catchment area. Then, we advertised the study on the local TV and in newspapers, and added gas coupons as reimbursement for time lost. Subsequently, we proposed conducting the interview in the subjects' homes, and added a letter endorsing the study signed by the family physician. These efforts achieved a response rate of 48.9%. To increase the participation rate further, we consulted with one of the largest market research companies in Italy, and implemented the following procedures: we altered the layout of the invitation letter, established a toll-free phone number through which potential participants could obtain study information, added to our invitation a letter from the mayor of Milan supporting our research project, and requested that family physicians call the subjects directly to inform them about the seriousness and scientific value of the study. We also provided a token of gratitude (gas coupon) to the physicians. With these measures, we achieved an acceptable response rate of 72%. Overall, in the pilot studies, we collected data on approximately 300 subjects. This level of response remained constant through the course of the full-scale study.

### Impact of incentives on participation rate and enrolled subjects' characteristics

After study completion, we assessed the impact of the involvement of the family physician in controls' response rate. Controls contacted by their family physician had a much higher participation rate (80.1%) than those (49.3%) not contacted.

To further evaluate the impact of incentives on study participation, we explored the socio-demographic differences between control subjects who were enrolled with few or no incentives (~49% response rate) and those who were enrolled with the improved procedures (~73% response rate). We included in these analyses all controls recruited during the pilot studies and those recruited in the main study up to March 2003 (N = 748). We found some suggestive associations: the high incentive group exhibited an increased family history of lung cancer (p = 0.03); in addition, borderline associations were observed for: awareness of the link between smoking and lung cancer (lower, p = 0.07), anxiety score (lower, p = 0.08) and depression score (higher, p = 0.13) as measured by the Hospital Anxiety and Depression Scale (HADS), intention to quit smoking (lower, p = 0.10), history of quit attempts (lower, p = 0.11), military service (lower frequency, p = 0.11), and percentage attending college (lower, p = 0.12). Adjustment for age, gender, and, when appropriate, smoking, did not substantially alter these findings. We also found small non-significant differences by incentive group in a panel of 15 short tandem repeat (STR) loci used to identify genetic differences between samples for quality control of sample handling and processing [13]. These analyses suggest that in studies with low response rates, estimates may be influenced by factors such as family history, education or behavioral characteristics.

In the pilot studies, we could not verify the efficacy of each incentive or procedure separately because different types of incentives were often offered together. We did, however, ask participants to rank the factors that influenced their participation. Among the most influential factors reported by subjects recruited through December 2003, "desire to help medical research" (78%), "reassured by the family physician" (53%), and "possibility to participate from home" (44%) were the most frequent "very high" scores. "Receiving compensation", "obtaining information by calling a toll-free number", and "receiving the letter from the mayor of Milan" were the factors with the most frequent, "very low" scores (61%, 47%, and 40%, respectively). The majority of subjects were not aware of the advertisements about the study that appeared on the local TV or newspapers. These data are relevant to future studies, with the caveat that they are based on self-reporting in one cultural setting, and need to be evaluated by direct comparison.

### Epidemiological and clinical data collection

Extensive epidemiological data have been collected through both a Computer Assisted Personal Interview (CAPI) to capture the major risk factors for lung cancer and a self-administered questionnaire to address behavioral aspects possibly associated with smoking persistence and diet (questionnaires are available on the EAGLE website). In particular, data on tobacco smoking included information on number of cigarettes, cigars, pipes, and cigarillos per day averaged over lifetime and in the last year, age at initiation/quit, quitting attempts and time between attempts, inhalation habits, cigarette/cigar brand, passive smoking during childhood, at workplace and at home during adulthood. Moreover, we collected data on tobacco smoking in first-degree relatives. To explore the determinants of smoking persistence we also added key behavioral rating scales including the Fagerström Test for Nicotine Dependence (FTND) [14], nicotine withdrawal [15], knowledge about smoking effects, Beck's Depression Inventory, Hospital Anxiety and Depression Scale (HADS) [16], alcohol dependence, Attention Deficit Disorder (ADD), and the Short-Form Revised Eysenck Personality Questionnaire [17]. A limited food frequency questionnaire evaluated diet for specific variables of interest: vegetables, fresh and dry fruit, ham, salami, and other processed meats, red and white meat consumption (with questions about meat cooking practices), pizza, pasta, alcohol, and vitamin/mineral supplements. Additionally, subjects were asked whether they were on special diets and for what reason. From each lung cancer case we also collected extensive clinical data, including histology and grading (ICD-O codes), TNM/stage (clinical and surgical, AJCC and UICC), imaging and pathology (surgery, biopsy, and cytology) reports, blood count and serum tumor markers, chemotherapy or radiation therapy for previous tumors, blood transfusions, and previous lung diseases with spirometry indexes. From approximately 10% of the cases, histology slides were scanned and digital images stored in a large database for archival, research, and educational purposes.

One of our goals is to integrate genomic and epidemiological data with clinical data on therapy outcome and survival in order to identify genetic factors that affect these factors. We are currently collecting data from cases on surgical procedures, chemotherapy (type, doses, duration, cycles, and breaks), radiation therapy (type, duration, dose, equipments, and breaks), major toxicities, ECOG performance status, recurrence, smoking after lung cancer diagnosis, vital status through the Vital Statistic Office, and death certificates through the Local Health Units (causes of death are coded following the ICD IX).

### Biospecimen collection

Specific laboratory Standard Operating Procedures were developed (and updated as warranted) within EAGLE to ensure quality control of every step involved in biospecimen collection, processing, transportation, tracking, shipping, and eventual long term storage. Approximately 90% of cases and 87% of controls donated a blood sample, and 7% of cases and 13% of controls donated buccal rinse samples (Table 1). Blood samples were transported from each hospital to the central laboratory within four hours of phlebotomy by a transportation team established *ad hoc *for EAGLE. Blood specimens were processed to obtain cryopreserved lymphocytes, RBC, granulocytes, DNA, RNA, whole blood, buffy coat, serum, plasma, and blood cards. For RNA collection and extraction, we also used PAX tubes (Paxgene Blood RNA System), which contain a solution that inhibits RNA degradation and gene induction as blood is drawn into the tube. Buccal cells obtained by mouthwash were processed to obtain DNA.

Lung tissue paraffin blocks and slides were collected from cases that underwent surgery, biopsy, or cytological examination of the lung tumor (Table 1). Multiple fresh "normal" lung tissue (adjacent and distant from the malignant lesion) and tumor samples, frozen in liquid nitrogen within 20 minutes from excision at surgery, were also collected from 436 cases (about 46% of the surgical cases). All biospecimens and accompanying forms were labeled using 2-D bar codes. Biospecimens were shipped according to international regulations on alternate weeks following different procedures based on biospecimen type, and tracked through a database [18] that stores detailed information on sample descriptions, dates, sample transfers, aliquoting, freezer locations, and material type that is linked to the repository for easy access and exchange of laboratory information.

### Data management

The study coordination center for EAGLE was established at the Epidemiology Research Center (EPOCA) of the University of Milan. Ancillary facilities included: 1) a Study Document Center for the collection and completeness verification of the computer-assisted questionnaire, and the scanning and verification of the optical readable forms and self-administered questionnaires; 2) a Storage and Processing Laboratory for the collection, processing, storage, and shipping of biospecimens; 3) a Data Processing Center, for the collection of all data in a central relational database (MS SQLServer). Data on subjects' accrual and data/biospecimen collection were regularly transmitted to the principal investigators at the National Cancer Institute (NCI), Bethesda, MD through automatically generated weekly reports. We routinely validated received data by: comparing information from different sources; assessing variable range and distribution; evaluating the quality of biospecimens through specific analyses conducted at the NCI laboratories on random samples; comparing numbers of cases accrued and those reported in the discharge records of the hospitals during the same time period to ensure that all consecutive cases were approached for the study; and verifying completeness of the database through multiple queries. Upon study completion, we developed a portal [19] for exchange of data, documents, timelines, meeting minutes, procedures, and draft manuscripts among investigators involved in the EAGLE data analyses, and a website [8] for public access to study design, collaborators, descriptive statistics, and publications.

Epidemiological analyses of these and other risk factors are ongoing, exploiting the richness of molecular data and the integrative approach described above. For example, the first analysis of gene expression changes due to tobacco smoking was recently completed [20].

## Discussion

Our study design anticipates configuring all the diverse classes of data described to address the biological and clinical challenges posed by lung cancer. We call our approach *integrative *to emphasize the inclusion of behavior (e.g., nicotine dependency, smoking intensity, depression, anxiety, and other psychological traits) and outcome (e.g., survival from lung cancer) with the traditional molecular epidemiology framework which uses biomarkers to elucidate the biological relationships between exposure, genes, and diseases. Moreover, the integrative approach allows cross-sectional analyses of multiple factors (e.g., germline genetic variation, somatic mutations and gene expression in relation to lung cancer risk or progression) [21]. This approach provides several key advantages over more fragmented designs: 1) It is highly efficient and cost effective, since information collected for one purpose can be leveraged for another, instead of each goal requiring independent planning, infrastructure and data collection; 2) Because the design includes diverse study domains, diverse questions can be addressed that are inaccessible to more constricted designs. For example, depression (a behavior) is known to be related to smoking (an exposure). However, only an integrated design can establish whether it is also related to lung cancer risk (taking into account smoking), or lung cancer survival (taking into account other prognostic factors), or whether genes related to smoking or depression are the same ones that influence lung cancer risk [22]. Through the parallel use of high technology approaches applied to tissue samples, similar questions can be addressed on the molecular level. For example, we may assess a gene through a chromosomal region, polymorphic variant, expression or methylation pattern, or its protein product. Eventually all these approaches can be combined to assess cancer networks; 3) The integrated approach can identify genes or other factors that span multiple stages in the development of disease to its denouement. In general, the major question of whether the same genes that contribute to the 'cause' of a cancer also stimulate its progression has been generally sidestepped by traditional epidemiological study designs. 4) It provides a study crucible where scientists of diverse disciplines can work in concert to forge a deeper interdisciplinary understanding of the disease etiology.

While tobacco consumption peaked in the United States in the second third of the 20^th ^century and somewhat later in Western Europe, worldwide per capita consumption continues to rise and therefore understanding the precise molecular basis and susceptibility factors associated with tobacco carcinogenesis remains a high priority as current prevention, screening and treatment approaches are all inadequate.

### Opportunities to collaborate

We designed EAGLE knowing that much of its value would arise from involvement with other investigators, individually and within consortia. EAGLE is already part of the International Lung Cancer Consortium (ILCCO) [23] and is open for collaboration with interested investigators. Given the population-based design, the rigorous enrollment and quality control strategies, the high response rate in both lung cancer cases and controls, the detailed epidemiological data, the excellent quality of clinical information and diagnostic procedures, and the ever-growing collection of molecular data, EAGLE will provide a superb framework for diverse studies and collaborative efforts, of which those listed above are just initial examples.

The EAGLE investigators also want to provide public access to the data, consistent with appropriate measures to protect confidentiality. Proposals from outside the study team for research projects to test specific hypotheses within EAGLE will be reviewed by an Advisory Board with rotating membership. Mechanisms and policies to enable data sharing to interested investigators are posted on the EAGLE website.

In conclusion, EAGLE is a new large population-based case-control study that explores the full spectrum of lung cancer etiology, from smoking addiction to lung cancer outcome, through cross examination of epidemiological, molecular, and clinical data. A sharper and more comprehensive vision of the complex nature of this disease is poised to accelerate the emergence of new preventive and therapeutic strategies with potentially enormous impact on public health.

## Abbreviations

ADD: Attention Deficit Disorder; AJCC: American Joint Committee on Cancer; CAPI: Computer Assisted Personal Interview; CT: Computed Tomography; MRI: Magnetic Resonance Imaging; DNA: Deoxyribonucleic Acid; ECOG: Eastern Cooperative Oncology Group; EPOCA: Epidemiology Research Center, University of Milan; FTND: Fagerström Test for Nicotine Dependence; HADS: Hospital Anxiety and Depression Scale; ICD IX: International Classification of Disease IX; ICD-O: International Classification of Diseases for Oncology; ILCCO: International Lung Cancer Consortium; IRB: Institutional Review Board; ISDN: Integrated Services Digital Network; NIH: National Institutes of Health; NCI: National Cancer Institute; PET: Positron Emission Tomography; RBC: Red Blood Cells; RNA: Ribonucleic Acid; SEER: Surveillance Epidemiology and End Results; TNM: Classification of Malignant Tumors; UICC: Union Internationale Contre le Cancer; WHO: World Health Organization.

## Competing interests

The authors declare that they have no competing interests

## Authors' contributions

MTL was responsible for the conception and design of this study and the study management, oversaw all aspects of the study including patients and controls recruitment, funding, quality control of the data, data analysis and interpretation, and manuscript writing; DC participated in the design and management of the study, and designed and implemented all the infrastructures for lung cancer cases' enrollment, and related weekly reports and quality control procedures; MRo participated in all study analyses and database implementation; AWB participated in the conception and design of the study, laboratory procedures, analytical strategies and questionnaire on behavioral traits; AMG, JHL, LG and MA participated in the study design, analytical strategies, and management, and verified the quality control of each procedural step; GM participated in the study design and development of the material for the collection of data on smoking exposure; AFS participated in the study design and developed the food-frequency questionnaire; IL reviewed pathological material to confirm the diagnosis of lung cancer and histologic classification; FP designed and developed the database and portal for the study; MC and MRu coordinated all field activities and developed related readable forms and infrastructures for data processing; BM and BA participated in the design of the laboratory component of the study, and directed laboratory procedures for the subjects' biospecimen collection, processing, storage, and shipments; AC participated in the design of the study, was responsible for all laboratory activities, and developed the Standard Operating Procedures for the laboratory; MT and SW participated in the study design, management and analytical strategies, and oversaw all steps of the study; ACP participated in the design and management of the study, and designed and implemented all the infrastructures for population controls' enrollment, and related weekly reports and quality control procedures; NEC was responsible for the conception and design of the study, and oversaw all aspects of the study activities, patients recruitment, funding, and interpretation of the data; PAB contributed to the study design, analytical strategy, and management, and was the ultimate responsible for all study activities at EPOCA, University of Milan.

## Pre-publication history

The pre-publication history for this paper can be accessed here:


